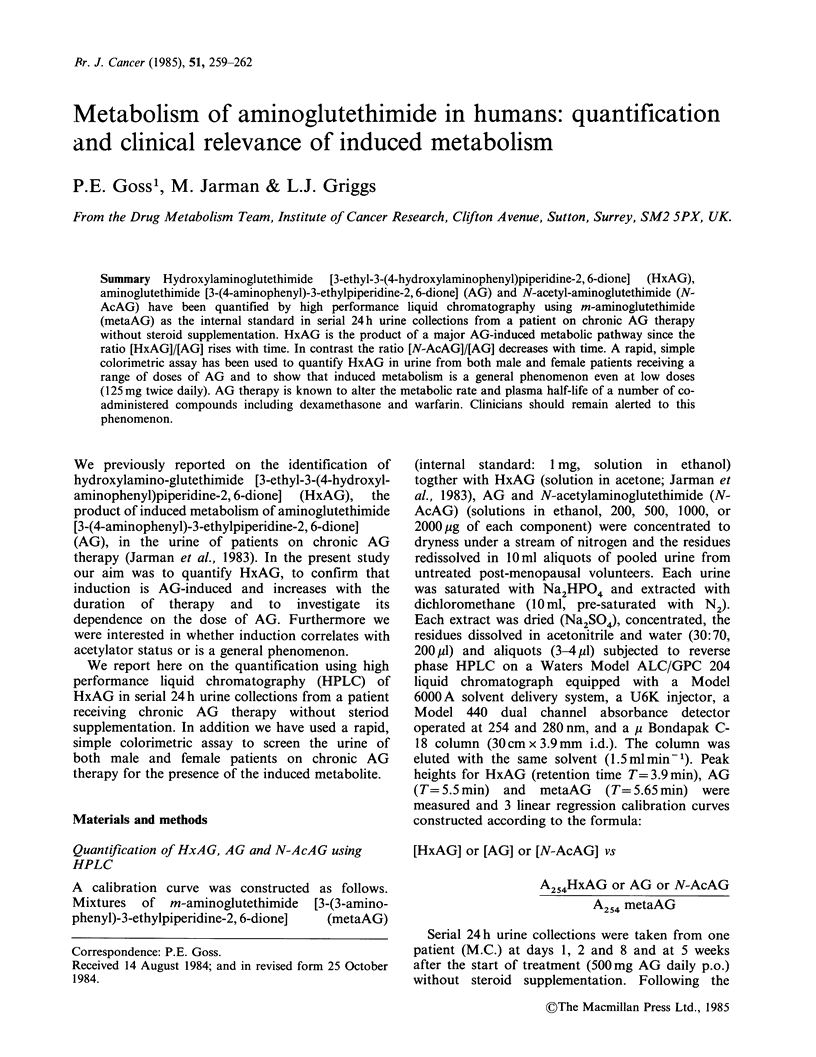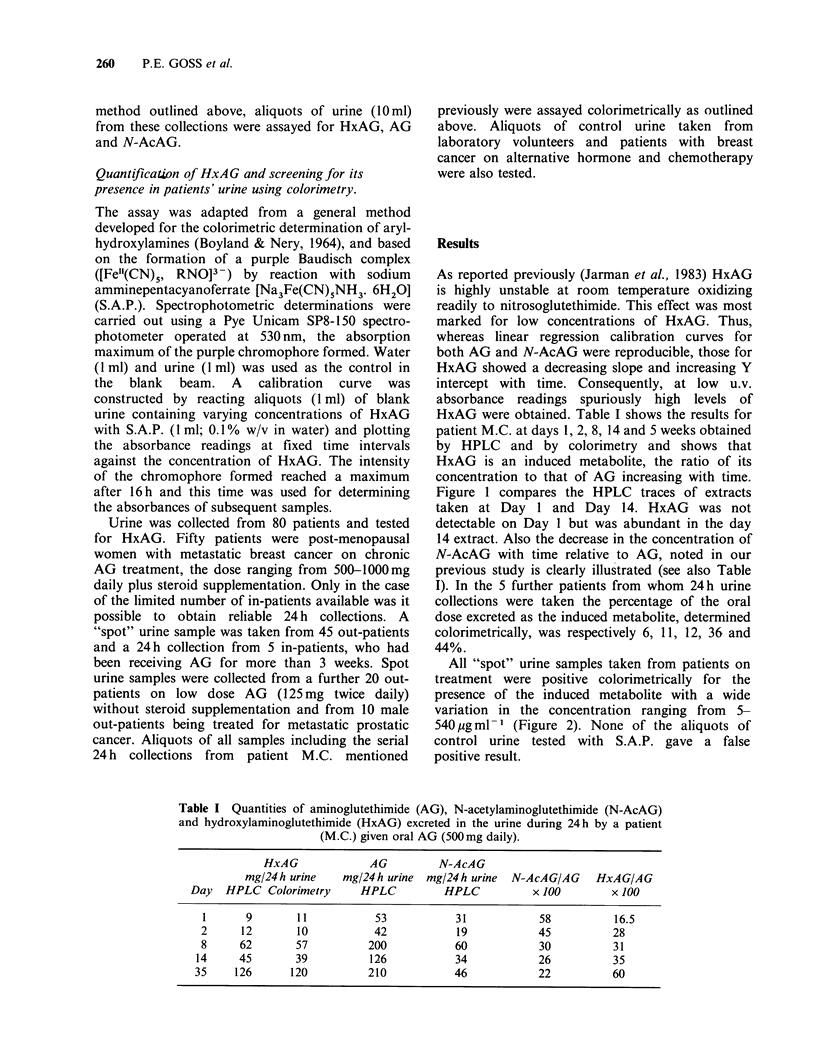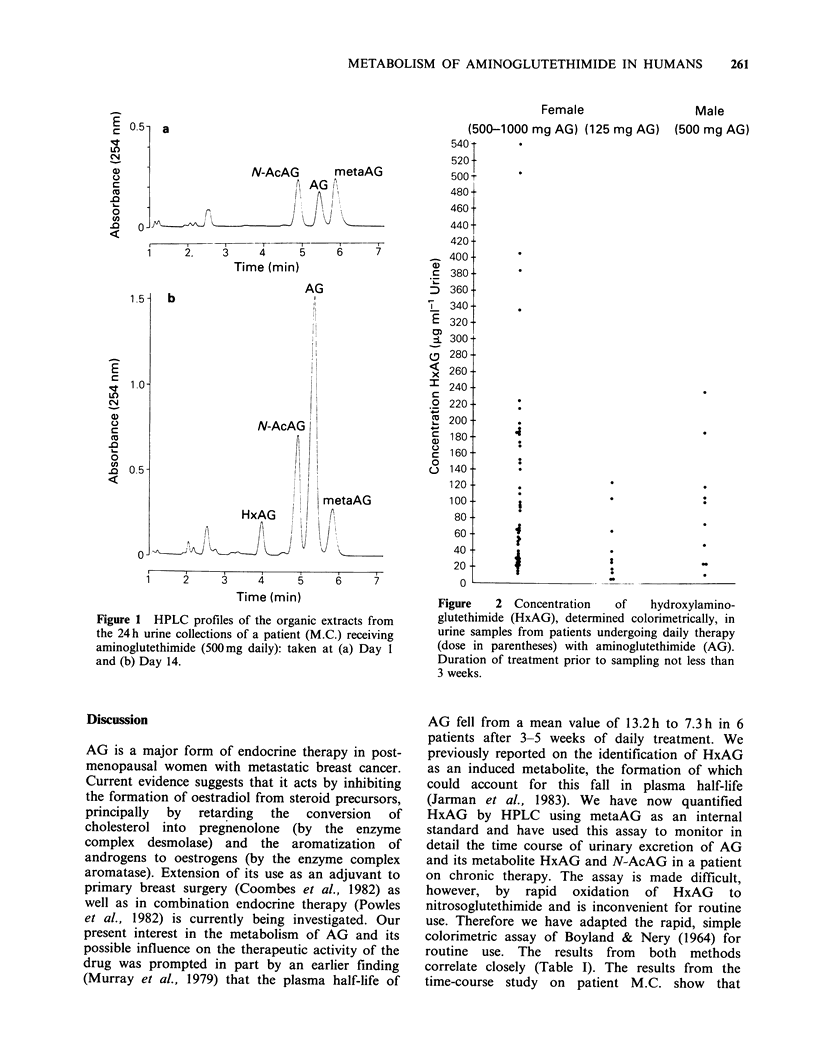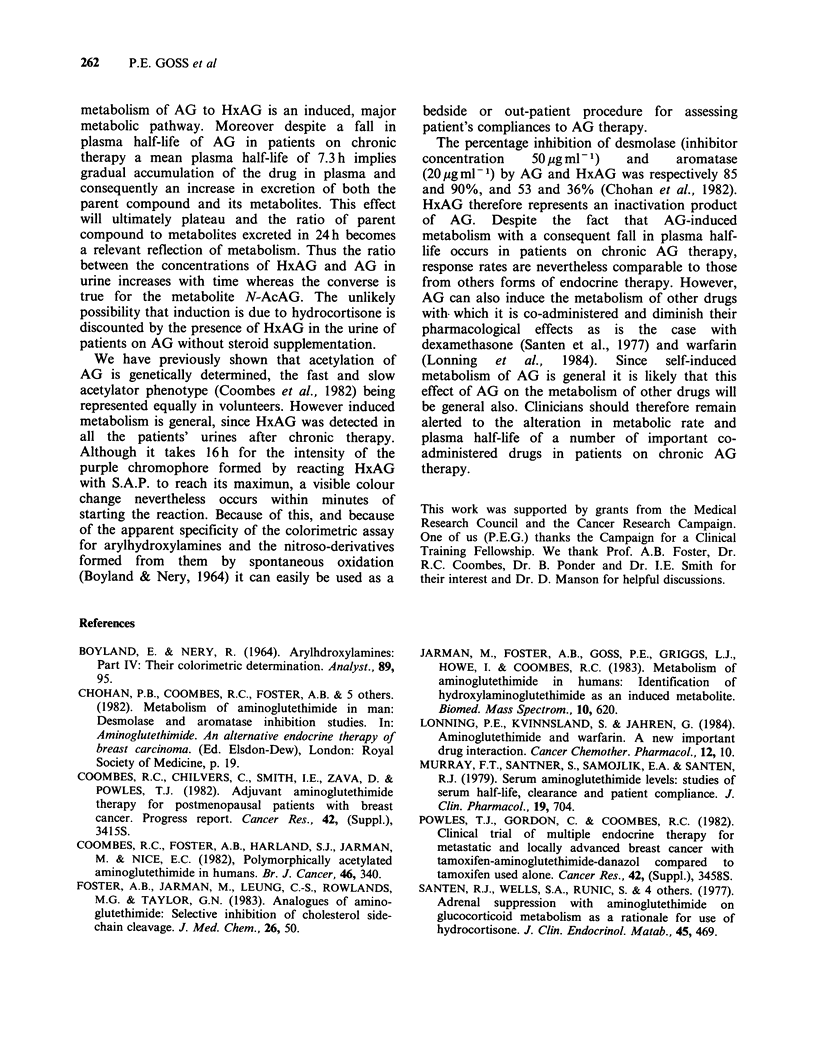# Metabolism of aminoglutethimide in humans: quantification and clinical relevance of induced metabolism.

**DOI:** 10.1038/bjc.1985.37

**Published:** 1985-02

**Authors:** P. E. Goss, M. Jarman, L. J. Griggs

## Abstract

Hydroxylaminoglutethimide [3-ethyl-3-(4-hydroxylaminophenyl)piperidine-2,6-dione] (HxAG), aminoglutethimide [3-(4-aminophenyl)-3-ethylpiperidine-2,6-dione] (AG) and N-acetyl-aminoglutethimide (N-AcAG) have been quantified by high performance liquid chromatography using m-aminoglutethimide (metaAG) as the internal standard in serial 24 h urine collections from a patient on chronic AG therapy without steroid supplementation. HxAG is the product of a major AG-induced metabolic pathway since the ratio [HxAG]/[AG] rises with time. In contrast the ratio [N-AcAG]/[AG] decreases with time. A rapid, simple colorimetric assay has been used to quantify HxAG in urine from both male and female patients receiving a range of doses of AG and to show that induced metabolism is a general phenomenon even at low doses (125 mg twice daily). AG therapy is known to alter the metabolic rate and plasma half-life of a number of coadministered compounds including dexamethasone and warfarin. Clinicians should remain alerted to this phenomenon.


*Br. J. Cancer* (1985), **51**, 259–262

### Metabolism of aminoglutethimide in humans: quantification and clinical relevance of induced metabolism

P.E. Goss[1], M. Jarman & L.J. Griggs

*From the Drug Metabolism Team, Institute of Cancer Research, Clifton Avenue, Sutton, Surrey, SM2 5PX, UK.*

**Summary** Hydroxylaminoglutethimide [3-ethyl-3-(4-hydroxylaminophenyl)piperidine-2, 6-dione] (HxAG), aminoglutethimide [3-(4-aminophenyl)-3-ethylpiperidine-2, 6-dione] (AG) and *N*-acetyl-aminoglutethimide (*N*-AcAG) have been quantified by high performance liquid chromatography using *m*-aminoglutethimide (metaAG) as the internal standard in serial 24 h urine collections from a patient on chronic AG therapy without steroid supplementation. HxAG is the product of a major AG-induced metabolic pathway since the ratio [HxAG]/[AG] rises with time. In contrast the ratio [*N*-AcAG]/[AG] decreases with time. A rapid, simple colorimetric assay has been used to quantify HxAG in urine from both male and female patients receiving a range of doses of AG and to show that induced metabolism is a general phenomenon even at low doses (125 mg twice daily). AG therapy is known to alter the metabolic rate and plasma half-life of a number of co-administered compounds including dexamethasone and warfarin. Clinicians should remain alerted to this phenomenon.

We previously reported on the identification of hydroxylamino-glutethimide [3-ethyl-3-(4-hydroxyl-aminophenyl)piperidine-2, 6-dione] (HxAG), the product of induced metabolism of aminoglutethimide [3-(4-aminophenyl)-3-ethylpiperidine-2, 6-dione] (AG), in the urine of patients on chronic AG therapy (Jarman *et al.*, 1983). In the present study our aim was to quantify HxAG, to confirm that induction is AG-induced and increases with the duration of therapy and to investigate its dependence on the dose of AG. Furthermore we were interested in whether induction correlates with acetylator status or is a general phenomenon.

We report here on the quantification using high performance liquid chromatography (HPLC) of HxAG in serial 24 h urine collections from a patient receiving chronic AG therapy without steriod supplementation. In addition we have used a rapid, simple colorimetric assay to screen the urine of both male and female patients on chronic AG therapy for the presence of the induced metabolite.

### Materials and methods

### *Quantification of HxAG, AG and N-AcAG using HPLC*

A calibration curve was constructed as follows. Mixtures of *m*-aminoglutethimide [3-(3-amino-phenyl)-3-ethylpiperidine-2, 6-dione] (metaAG) (internal standard: 1 mg, solution in ethanol) togther with HxAG (solution in acetone; Jarman *et al.*, 1983), AG and *N*-acetylaminoglutethimide (*N*-AcAG) (solutions in ethanol, 200, 500, 1000, or 2000 $\mu$g of each component) were concentrated to dryness under a stream of nitrogen and the residues redissolved in 10 ml aliquots of pooled urine from untreated post-menopausal volunteers. Each urine was saturated with $Na_2HPO_4$ and extracted with dichloromethane (10 ml, pre-saturated with $N_2$). Each extract was dried ($Na_2SO_4$), concentrated, the residues dissolved in acetonitrile and water (30:70, 200 $\mu$l) and aliquots (3–4 $\mu$l) subjected to reverse phase HPLC on a Waters Model ALC/GPC 204 liquid chromatograph equipped with a Model 6000 A solvent delivery system, a U6K injector, a Model 440 dual channel absorbance detector operated at 254 and 280 nm, and a $\mu$ Bondapak C-18 column (30 cm × 3.9 mm i.d.). The column was eluted with the same solvent (1.5 ml min$^{-1}$). Peak heights for HxAG (retention time $T=3.9$ min), AG ($T=5.5$ min) and metaAG ($T=5.65$ min) were measured and 3 linear regression calibration curves constructed according to the formula:

$$[\text{HxAG}] \text{ or } [\text{AG}] \text{ or } [N\text{-AcAG}] \ vs \ \frac{A_{254}\text{HxAG or AG or } N\text{-AcAG}}{A_{254}\text{ metaAG}}$$

Serial 24 h urine collections were taken from one patient (M.C.) at days 1, 2 and 8 and at 5 weeks after the start of treatment (500 mg AG daily p.o.) without steroid supplementation. Following the

Correspondence: P.E. Goss.

Received 14 August 1984; and in revised form 25 October 1984.

©The Macmillan Press Ltd., 1985

method outlined above, aliquots of urine (10 ml) from these collections were assayed for HxAG, AG and *N*-AcAG.

*Quantification of HxAG and screening for its presence in patients' urine using colorimetry.*

The assay was adapted from a general method developed for the colorimetric determination of arylhydroxylamines (Boyland & Nery, 1964), and based on the formation of a purple Baudisch complex ($[Fe^{II}(CN)_5, RNO]^{3-}$) by reaction with sodium amminepentacyanoferrate $[Na_3Fe(CN)_5NH_3. 6H_2O]$ (S.A.P.). Spectrophotometric determinations were carried out using a Pye Unicam SP8-150 spectrophotometer operated at 530 nm, the absorption maximum of the purple chromophore formed. Water (1 ml) and urine (1 ml) was used as the control in the blank beam. A calibration curve was constructed by reacting aliquots (1 ml) of blank urine containing varying concentrations of HxAG with S.A.P. (1 ml; 0.1% w/v in water) and plotting the absorbance readings at fixed time intervals against the concentration of HxAG. The intensity of the chromophore formed reached a maximum after 16 h and this time was used for determining the absorbances of subsequent samples.

Urine was collected from 80 patients and tested for HxAG. Fifty patients were post-menopausal women with metastatic breast cancer on chronic AG treatment, the dose ranging from 500–1000 mg daily plus steroid supplementation. Only in the case of the limited number of in-patients available was it possible to obtain reliable 24 h collections. A "spot" urine sample was taken from 45 out-patients and a 24 h collection from 5 in-patients, who had been receiving AG for more than 3 weeks. Spot urine samples were collected from a further 20 out-patients on low dose AG (125 mg twice daily) without steroid supplementation and from 10 male out-patients being treated for metastatic prostatic cancer. Aliquots of all samples including the serial 24 h collections from patient M.C. mentioned previously were assayed colorimetrically as outlined above. Aliquots of control urine taken from laboratory volunteers and patients with breast cancer on alternative hormone and chemotherapy were also tested.

### Results

As reported previously (Jarman *et al.*, 1983) HxAG is highly unstable at room temperature oxidizing readily to nitrosoglutethimide. This effect was most marked for low concentrations of HxAG. Thus, whereas linear regression calibration curves for both AG and *N*-AcAG were reproducible, those for HxAG showed a decreasing slope and increasing Y intercept with time. Consequently, at low u.v. absorbance readings spuriously high levels of HxAG were obtained. Table I shows the results for patient M.C. at days 1, 2, 8, 14 and 5 weeks obtained by HPLC and by colorimetry and shows that HxAG is an induced metabolite, the ratio of its concentration to that of AG increasing with time. Figure 1 compares the HPLC traces of extracts taken at Day 1 and Day 14. HxAG was not detectable on Day 1 but was abundant in the day 14 extract. Also the decrease in the concentration of *N*-AcAG with time relative to AG, noted in our previous study is clearly illustrated (see also Table I). In the 5 further patients from whom 24 h urine collections were taken the percentage of the oral dose excreted as the induced metabolite, determined colorimetrically, was respectively 6, 11, 12, 36 and 44%.

All "spot" urine samples taken from patients on treatment were positive colorimetrically for the presence of the induced metabolite with a wide variation in the concentration ranging from 5–540 $\mu g\,ml^{-1}$ (Figure 2). None of the aliquots of control urine tested with S.A.P. gave a false positive result.

**Table I** Quantities of aminoglutethimide (AG), N-acetylaminoglutethimide (N-AcAG) and hydroxylaminoglutethimide (HxAG) excreted in the urine during 24 h by a patient (M.C.) given oral AG (500 mg daily).

| | *HxAG mg/24 h urine* | | *AG mg/24 h urine* | *N-AcAG mg/24 h urine* | | |
|---|---|---|---|---|---|---|
| *Day* | *HPLC* | *Colorimetry* | *HPLC* | *HPLC* | *N-AcAG/AG × 100* | *HxAG/AG × 100* |
| 1 | 9 | 11 | 53 | 31 | 58 | 16.5 |
| 2 | 12 | 10 | 42 | 19 | 45 | 28 |
| 8 | 62 | 57 | 200 | 60 | 30 | 31 |
| 14 | 45 | 39 | 126 | 34 | 26 | 35 |
| 35 | 126 | 120 | 210 | 46 | 22 | 60 |

**Figure 1** HPLC profiles of the organic extracts from the 24 h urine collections of a patient (M.C.) receiving aminoglutethimide (500 mg daily): taken at (a) Day 1 and (b) Day 14.

**Figure 2** Concentration of hydroxylamino-glutethimide (HxAG), determined colorimetrically, in urine samples from patients undergoing daily therapy (dose in parentheses) with aminoglutethimide (AG). Duration of treatment prior to sampling not less than 3 weeks.

### Discussion

AG is a major form of endocrine therapy in post-menopausal women with metastatic breast cancer. Current evidence suggests that it acts by inhibiting the formation of oestradiol from steroid precursors, principally by retarding the conversion of cholesterol into pregnenolone (by the enzyme complex desmolase) and the aromatization of androgens to oestrogens (by the enzyme complex aromatase). Extension of its use as an adjuvant to primary breast surgery (Coombes *et al.*, 1982) as well as in combination endocrine therapy (Powles *et al.*, 1982) is currently being investigated. Our present interest in the metabolism of AG and its possible influence on the therapeutic activity of the drug was prompted in part by an earlier finding (Murray *et al.*, 1979) that the plasma half-life of AG fell from a mean value of 13.2 h to 7.3 h in 6 patients after 3–5 weeks of daily treatment. We previously reported on the identification of HxAG as an induced metabolite, the formation of which could account for this fall in plasma half-life (Jarman *et al.*, 1983). We have now quantified HxAG by HPLC using metaAG as an internal standard and have used this assay to monitor in detail the time course of urinary excretion of AG and its metabolite HxAG and *N*-AcAG in a patient on chronic therapy. The assay is made difficult, however, by rapid oxidation of HxAG to nitrosoglutethimide and is inconvenient for routine use. Therefore we have adapted the rapid, simple colorimetric assay of Boyland & Nery (1964) for routine use. The results from both methods correlate closely (Table I). The results from the time-course study on patient M.C. show that metabolism of AG to HxAG is an induced, major metabolic pathway. Moreover despite a fall in plasma half-life of AG in patients on chronic therapy a mean plasma half-life of 7.3 h implies gradual accumulation of the drug in plasma and consequently an increase in excretion of both the parent compound and its metabolites. This effect will ultimately plateau and the ratio of parent compound to metabolites excreted in 24 h becomes a relevant reflection of metabolism. Thus the ratio between the concentrations of HxAG and AG in urine increases with time whereas the converse is true for the metabolite *N*-AcAG. The unlikely possibility that induction is due to hydrocortisone is discounted by the presence of HxAG in the urine of patients on AG without steroid supplementation.

We have previously shown that acetylation of AG is genetically determined, the fast and slow acetylator phenotype (Coombes *et al.*, 1982) being represented equally in volunteers. However induced metabolism is general, since HxAG was detected in all the patients' urines after chronic therapy. Although it takes 16 h for the intensity of the purple chromophore formed by reacting HxAG with S.A.P. to reach its maximun, a visible colour change nevertheless occurs within minutes of starting the reaction. Because of this, and because of the apparent specificity of the colorimetric assay for arylhydroxylamines and the nitroso-derivatives formed from them by spontaneous oxidation (Boyland & Nery, 1964) it can easily be used as a bedside or out-patient procedure for assessing patient's compliances to AG therapy.

The percentage inhibition of desmolase (inhibitor concentration $50\,\mu g\,ml^{-1}$) and aromatase ($20\,\mu g\,ml^{-1}$) by AG and HxAG was respectively 85 and 90%, and 53 and 36% (Chohan *et al.*, 1982). HxAG therefore represents an inactivation product of AG. Despite the fact that AG-induced metabolism with a consequent fall in plasma half-life occurs in patients on chronic AG therapy, response rates are nevertheless comparable to those from others forms of endocrine therapy. However, AG can also induce the metabolism of other drugs with which it is co-administered and diminish their pharmacological effects as is the case with dexamethasone (Santen et al., 1977) and warfarin (Lonning *et al.*, 1984). Since self-induced metabolism of AG is general it is likely that this effect of AG on the metabolism of other drugs will be general also. Clinicians should therefore remain alerted to the alteration in metabolic rate and plasma half-life of a number of important co-administered drugs in patients on chronic AG therapy.

This work was supported by grants from the Medical Research Council and the Cancer Research Campaign. One of us (P.E.G.) thanks the Campaign for a Clinical Training Fellowship. We thank Prof. A.B. Foster, Dr. R.C. Coombes, Dr. B. Ponder and Dr. I.E. Smith for their interest and Dr. D. Manson for helpful discussions.

### References

BOYLAND, E. & NERY, R. (1964). Arylhdroxylamines: Part IV: Their colorimetric determination. *Analyst.*, **89,** 95.

CHOHAN, P.B., COOMBES, R.C., FOSTER, A.B. & 5 others. (1982). Metabolism of aminoglutethimide in man: Desmolase and aromatase inhibition studies. In: *Aminoglutethimide. An alternative endocrine therapy of breast carcinoma.* (Ed. Elsdon-Dew), London: Royal Society of Medicine, p. 19.

COOMBES, R.C., CHILVERS, C., SMITH, I.E., ZAVA, D. & POWLES, T.J. (1982). Adjuvant aminoglutethimide therapy for postmenopausal patients with breast cancer. Progress report. *Cancer Res.*, **42,** (Suppl.), 3415S.

COOMBES, R.C., FOSTER, A.B., HARLAND, S.J., JARMAN, M. & NICE, E.C. (1982), Polymorphically acetylated aminoglutethimide in humans. *Br. J. Cancer*, **46,** 340.

FOSTER, A.B., JARMAN, M., LEUNG, C.-S., ROWLANDS, M.G. & TAYLOR, G.N. (1983). Analogues of amino-glutethimide: Selective inhibition of cholesterol side-chain cleavage. *J. Med. Chem.*, **26,** 50.

JARMAN, M., FOSTER, A.B., GOSS, P.E., GRIGGS, L.J., HOWE, I. & COOMBES, R.C. (1983). Metabolism of aminoglutethimide in humans: Identification of hydroxylaminoglutethimide as an induced metabolite. *Biomed. Mass Spectrom.*, **10,** 620.

LONNING, P.E., KVINNSLAND, S. & JAHREN, G. (1984). Aminoglutethimide and warfarin. A new important drug interaction. *Cancer Chemother. Pharmacol.*, **12,** 10.

MURRAY, F.T., SANTNER, S., SAMOJLIK, E.A. & SANTEN, R.J. (1979). Serum aminoglutethimide levels: studies of serum half-life, clearance and patient compliance. *J. Clin. Pharmacol.*, **19,** 704.

POWLES, T.J., GORDON, C. & COOMBES, R.C. (1982). Clinical trial of multiple endocrine therapy for metastatic and locally advanced breast cancer with tamoxifen-aminoglutethimide-danazol compared to tamoxifen used alone. *Cancer Res.*, **42,** (Suppl.), 3458S.

SANTEN, R.J., WELLS, S.A., RUNIC, S. & 4 others. (1977). Adrenal suppression with aminoglutethimide on glucocorticoid metabolism as a rationale for use of hydrocortisone. *J. Clin. Endocrinol. Matab.*, **45,** 469.